# Association of Vascular Endothelial Growth Factor Polymorphisms with Asthma in Tunisian Children

**Published:** 2008-04-10

**Authors:** Jihene Lachheb, Hanene Chelbi, Imen Ben Dhifallah, Jamel Ammar, Kamel Hamzaoui, Agnès Hamzaoui

**Affiliations:** 1 Medicine University of Tunis, Homeostasis and cell Dysfunction Unit Research 99/UR/08-40, Tunis, Tunisia; 2 Pneumology Hospital A Mami, Pavillon B; Department of Pediatric and Respiratory Diseases, Ariana, Tunisia

**Keywords:** VEGF, polymorphisms, asthma

## Abstract

**Background:**

Previous studies demonstrated that the vascular endothelial growth factor (VEGF) was being implicated in the airways inflammation and remodeling process in patients with asthma.

**Aims:**

We explored the relationship of three polymorphisms in the VEGF gene with asthma in both case control and family studies.

**Methods:**

We Genotyped a total of 210 children with asthma, 224 unrelated controls and 160 parents for the +936 C >T (rs3025039), −634 G > C (rs2010963) and −2549 −2567 del 18 of the VEGF promoter region. The Mutations were identified with polymerase chain reaction followed by restriction fragment length polymorphism (RFLP) analysis for the +936 C > T, and −634 G > C polymorphisms.

**Results:**

Of the three polymorphisms studied, a borderline association with asthma was found for the G allele in the −634 G > C polymorphism (p = 0.059). No Statistically significant differences were observed for both +936 C > T, and −2549 −2567 del 18 polymorphisms between asthmatic patients and controls, considering either allelic or genotypic frequencies. The distribution of genotypes according to the severity status revealed a significant differences for the +936 C > T, and −2549 −2567 del 18 polymorphisms. In addition, association was found with the haplotypes inferred by the three polymorphisms and asthma susceptibility.

**Conclusion:**

We suggest that VEGF Gene polymorphisms can be implicated in asthma.

## Introduction

Vascular endothelial growth factor (VEGF) is a major angiogenic factor and is a prime regulator of endothelial cell proliferation. It plays a crucial role in physiological vasculogenesis and vascular permeability (Ferrara, 1992 and Yancopoulos, 2000) The VEGF gene expression is regulated by a variety of hormones, growth factors, lipopolysaccharide (LPS) (Watson, 2000) hypoxia (Neufeld, 1999), and cytokines (Ferrara, 1999), including, Platelet Derived Growth Factor (PDGF), Epidermal Growth Factor (EGF), Transforming Growth Factor β (TGF-b), Interleukine 1 (IL-1) and Interleukine 6 (IL-6) (Ferrara, 2003), suggesting that several other angiogenic factors may act by regulating VEGF expression (Klagsbrun, 1996). Increased VEGF expression resulting in inappropriate VEGF-induced angiogenesis is implicated in a number of disease pathologies. It is also widely expressed within many different highly vascularized organs, including the lung (Maniscalco, 1997). Compelling evidence suggests that mucosal neovascularisation occurs in chronic asthma (Li, 1997 and Salvato, 2001) and the VEGF level was increased in the airways of subjects with both acute (Hoshino, 2001) and stable (Lee, 2001) asthma. Studies have implicated growth factors including vascular VEGF and fibroblast growth factor 2 (FGF2) derived from eosinophils, macrophages and smooth muscle cells in its causation (Hoshino, 2001). Inverse correlation of mucosal vascularity with airway calibre (Salvato, 2001) suggests that neovascularisation may contribute to airways obstruction. It has been reported that both the number and percentage of vessels in bronchial mucosa taken from subjects with even mild asthma were higher than those in control subjects (Li, 1997; Kuwano, 1993; Chetta, 2005). There is increasing evidence to support a key role for VEGF, as a mediator of structural changes in the airway wall of asthmatics. Further the VEGF was produced by epithelial cells and preferentially by Th2 versus Th1 cells. In this setting, it had a critical role in Th2 inflammation, cytokine production and physiologic dysregulation of atopic disease (Lee, 2004).

The human VEGF gene is localized in chromosome 6p21.3 (Lutty, 1996) and comprises a 14-kb coding region organized in eight exons, separated by seven introns (Vincenti, 1996). This region of the genome (6p21) contains the major histocompatibility complex (MHC) and shows the strongest evidence for linkage to the asthma phenotype in most studies (wjst, 1999). The *VEGF* gene includes at least three polymorphisms that are relatively common and may influence VEGF expression. Two of these polymorphisms are located in the promoter region at −2549 −2567 del 18 and −634 G >C (rs2010963), relative to the translation start site, and have been associated with increased VEGF expression (Lambrechts, 2003 and Shahbazi, 2002) The third polymorphism located in the 3′ untranslated region (+936 C >T; rs3025039) is associated with substantially increased serum VEGF levels (Renner, 2000 and Krippl, 2003). Given that no data was shown to approve or to deny the implication of these polymorphisms with asthma disease, we further investigated the role of these polymorphisms in Tunisian asthmatic children.

## Material and Methods

### Subjects

#### Controls

The control group consisted of 224 children presenting to the emergency department of Tunis Children Hospital with other than respiratory or atopic complaints. To qualify as a control subjects, there should be no history of respiratory disease or asthma and no first degree relative with a history of asthma. All those controls did not suffer from any chronic disease and did not have any allergic disease (gave negative skin prick results, had normal total IgE level ≤200 UI/ml). Patients and controls belonged to the same ethnic group: African white Caucasian.

#### Patients

A total of 210 children (aged from 5 to 16 years, mean age 11.5) were enrolled in this study. The diagnosis and classification of the clinical severity of asthma was made according to the GINA guidelines.

This group of asthmatic children consisted of 5 subjects with intermittent asthma, 135 with mild persistent asthma, 60 with moderate persistent asthma, and 10 with severe persistent asthma. Atopic status was defined by a positive skin test reaction characterized by a wheal of 3mm in diameter, to a panel of common allergens in the presence of a positive histamine control and a negative uncoated control, and by measurement of total IgE level: positive values were taken as ≥200 IU/ml. (BECKMAN. Access Immunoassay system. FR).

Among the 210 asthmatic children there were 139 atopics and 71 non atopics who had negative skin test responses to common allergens (skin prick tests were performed by specially trained nurses with a panel of common allergens). In our study asthma was frequently associated with a heterogeneous group of clinical disorders including rhinitis, sinusitis and dermatitis. The clinical profiles are shown in table 1.

#### Families

Among the 210 asthmatic children, forty of them and their respective parents were also studied for linkage analyses.

All families were African white Caucasian and comprised the two parents and an affected child. Information on the prevalence of physician diagnosis of asthma in each family was ascertained by a questionnaire completed separately for each family member by the parents.

The study was approved by our National Ethics Committee and was conducted in accordance with the guidelines in the declaration of Helsinki. Informed consent was obtained from all parents of asthmatics and controls participating in this study.

## Genotyping of Polymorphisms Within the VEGF Gene

All asthmatics, their parents and controls were genotyped by polymerase chain reaction—based restriction fragment length polymorphism. For +936 C >T (rs3025039) and −634 G > C (rs2010963) mutations and a simple polymerase chain reaction for 2549 −2567 del 18 of the VEGF promoter region.

The Total volume of the PCR was 25 μl containing 100 ng of genomic DNA, 1× PCR- Buffer (Qiagen, Hilden, Germany), 0.5mM Of a dNTP- Mix (Fermentas), 0.5 Units of Taq DNA Polymerase (Fermentas) And 3 pmol of each primer (Biomers, Belgium). The Final MgCl2 Concentrations are 2, 3 and 4 respectively for the three polymorphisms. The primer used for +936 C > T are F: 5′-aaggaagaggagactctgcgcagagc-3′ R: 5′-taaatgtatgtatgtgggtgggtgtgtctacag-3′, for −634 G > C F: 5′-atttatttttgcttgccatt-3′ R: 5′-gtctgtctgtctgtccgtca-3′, and for 2549 −2567 del 18 are F: 5′-cctggagcgttttggttaaa-3′ R: 5′-atataggaagcagcttggaa-3′. The PCR Comprised an initial denaturation step (95°C for 15 min.), 35 Cycles (95°C for 30 s, primer annealing temperature (66°C for +936 C > T, 59°C for −634 G > C and 54°C for 2549 −2567 del 18 for 30 s), 72°C for 30 s and a final extension step (72°C For 10 min.). The Volume of the restriction assays was 25 μl containing 10 μl of the PCR product, the appropriate Buffer, and 5U of each restriction enzyme Hin1II and FaqI for +936 C > T and −634 G >C polymorphisms respectively (Fermentas). For the +936 C > T the assay thus yields two bands: 122 and 86 bp for TT genotype, three bands for CT genotype: 208, 122, and 86 bp and one 208 bp band for CC. For the −634 G > C the assay thus yields two bands: 193 and 111 bp for GG genotype, three bands for GC genotype: 193, 111, and 304 bp and one 304 bp band for CC genotype. For the 2549 −2567 del 18 the Fragment Sizes were 234 bp when the insertion of 18(bp) is present and 216 bp when the deletion of 18 (bp) is present.

## Statistical Analysis

Differences in alleles and genotypes frequencies between patients and healthy controls were compared using the standard Chi squared test (Epistat statistical package Epi Info version 6). When the expected cell number was less than 5, Fisher’s exact test was used. The strength of a gene association is indicated by the odds ratio (OR). The odds ratio and the 95% confidence intervals (CI) were calculated whenever applicable. Haplotypes analyses were determined based on a Bayesian algorithm using the phase program.

For the linkage analyses in family study, the TDT was used to test for transmission of alleles at a particular locus from heterozygous parents to their affected offspring (Spielman, 1993). The proportion of alleles transmitted from both mother and father was tested against a null hypothesis of 50% transmission by χ^2^ analysis. Probability values of 0.05 or less were regarded as statistically significant.

## Results

The association between asthma and VEGF polymorphisms (+936 C > T, −634 G > C and −2549 −2567 del 18) was investigated by determining the occurrence of each genotype and alleles in asthmatic and control children. No significant difference was detected between the patient and control groups in genotype distribution of any of the polymorphisms studied (p = 0.15) (p = 0.059) (p = 0.56) respectively for +936 C > T, −634 G > C, and for −2549 −2567 del 18 (Table 2). The results showed that alleles of neither the +936 C > T, nor −2549 −2567 del 18 were found to be associated with asthma. A slight borderline association was found with allele distribution of the −634 G > C polymorphisms (p = 0.059).

We also estimated haplotypes for the +936 C > T, −634 G > C and −2549 −2567 del 18 Haplotypes analyses on these polymorphisms revealed functionally distinct haplotypes associated with asthma (p > 10^−3^) (Table 3). There are total of seven common haplotypes among both cases and controls. The distribution of different haplotypes was not similar between asthmatic and control children. The Most common haplotypes were: the −634**C**/+936**C**/−2549**D**, with the frequencies of 32.14% and 33.25% and −634**G**/+936**C**/−2549**I** with the frequencies of 32.38% and 36.38% in respectively cases and controls. Two Haplotypes −634**G**/+936**T**/−2549**D**(p = 0.0004) and −634**C**/+936**T**/−2549**D** (p = 0.014) were associated with asthma including the last one which present significantly higher risk with an OR (95% CI) = 1.53 (1.07–2.18).

After applying the appropriate statistical analyses for each genetic variant, we did not observe any statistically significant deviation of the +936 C > T, −634 G > C and −2549 −2567 del 18 variants between atopic asthmatics and non atopic one (Table 4). The distribution of haplotype frequencies did not show any statistically significant differences between atopic and non atopic groups (data not shown).

A statistically significant difference was demonstrated for genotype distribution of the VEGF polymorphisms +936 C > T (p = 0.0001) and for −2549 −2567 del 18 (p = 0.02), when the intermittent/mild and moderate/severe asthmatics were compared (Table 5).

## Family study

Implicitly, we also assume that disease-susceptibility alleles are preferentially transmitted from parents to the affected offspring and that this effect can be captured by the transmission/disequilibrium test (TDT). We tested eighty families of asthmatic children for a possible association between asthma and VEGF polymorphisms. Only 65, 68 and 71 families respectively for the −634 G > C, +936 C > T and 2549 −2567 del 18 polymorphisms, on which we can apply for the TDT (Table 6). TDT analysis showed transmission of C, C, and D allele of respectively −634 G > C, +936 C > T and 2549 −2567 del 18 polymorphisms to offspring with asthma occurred more frequently than G, T and I allele but not significant (p = 0.17) (p = 0.22) (p = 0.09).

## Discussion

Asthma is a chronic inflammatory disease characterized by some common changes in asthmatic airway walls including growth and proliferation of new vessels (2002). Increased VEGF levels in induced sputum, (Asai, 2003; Asai, 2002; Kanazawa, 2002) bronchoalveolar lavage fluid, (Feltis, 2006) and VEGF-positive cells in bronchial biopsies (Hoshino, 2001) have been found in patients with asthma compared to healthy controls. The increased vascularity of bronchial mucosa in asthmatic subjects has been related to increased numbers of VEGF-positive cells, suggesting a pathogenic role for VEGF in the pathology of the asthmatic airway (Hoshino, 2001). In addition, VEGF has been related to increased basement membrane thickness in biopsies from asthmatic patients, suggesting a possible role of VEGF in airway remodeling (Chetta, 2005). Given the important role of VEGF in asthma we performed a genetic study concerning VEGF in asthma.

The present study is the first to be conducted on asthmatic children, there are no studies of asthma and VEGF variants have been performed. We tested for an association between markers in VEGF gene with asthma. Given the important role of VEGF in the remodeling process in asthma, we looked for a possible difference in the allele and genotype frequencies of three polymorphisms (+936 C > T, −634 G > C and −2549 −2567 del 18 asthmatic and control children. For −2549 −2567 del 18 of the VEGF promoter region, we found no evidence of association between this polymorphism and asthma, which was supposed to have functional relevance in VEGF promoter activity. When we compared the distribution of genotypes among the group of asthmatics according to the severity status we conclude that two of these polymorphisms +936 C > T, and −2549 −2567 del 18 are associated with the severity status. Although the specific role of this polymorphism in the VEGF gene has not yet been elucidated, it appears that the D allele increase VEGF expression, as assessed by the increased VEGF activity of transcription 1.95 fold compared to the I allele (Yang, 2003). In addition to this promoter region polymorphism, the C allele of a common polymorphism located in the 3′ untranslated region (+936 C > T) is known to be associated with substantially increased serum VEGF levels (Shahbazi, 2002; Renner, 2000). Carriers of the 936 T allele have been shown to have lower VEGF plasma levels than non-carriers (Shahbazi, 2002; Renner, 2000). The 936 C allele is one of the core sequences for the potential binding of Papillomavirus regulator E2 and the C to T change at position 936 results in the loss of the core binding sequence for this transcription factor. The third polymorphisms that we have studied is the −634 G > C that reported to was significantly correlated with lipopolysaccharide (LPS)-stimulated VEGF production from peripheral blood mononuclear cells (PBMCs) of healthy subjects, also highest production was observed for the GG genotype, the intermediate production for the CG genotype, and lowest production for the CC genotype (Watson, 2000). On the contrary to Awata et al. who found significantly higher VEGF levels in healthy subjects with the CC genotype than those with the other genotypes (Awata, 2002). In our study we didn’t find any association between theses polymorphisms and asthma when we analyze each polymorphism independently. Therefore it would be helpful to have the plausibility of combining these three polymorphisms. The global test of haplotype association was statistically significant (p> 10^−3^) and we suggest that −634**C**/+936**T**/−2549**D** haplotype was associated with much higher risk of having asthma than other haplotypes.

The frequency of the T allele at the +936 C > T untranslated region of VEGF was (24.3%) in Tunisian control population. This frequency was the highest compared with Italian population frequency (19.4%), the American population (14%), and Korean population (13.4%) (Boiardi, 2003; Jacobs, 2006; Han, 2004). The frequency of the G allele at the −634 promoter region of VEGF gene in the present study is (50.4%) less than the frequencies found in American, Italian and Korean samples (68%, 61%, 57%) (Boiardi, 2003; Jacobs, 2006; Han, 2004); which is more than frequency founded in Greek ethnicity (29.2%) (Papazoglou, 2004), suggesting the ethnic variability in the allelic frequencies of this gene. Because of this variation in gene frequencies between different ethnic groups, it is of importance to note that it’s crucial to generalize these findings when we try to establish the relationship between a polymorphic marker and asthma.

For the atopy part of this study, we did not find a significant statistically difference in the global distribution of genetic variants in spite the positive correlation found between VEGF and TH2 antigen sensitization (Lee, 2004) which imply that VEGF plays an important role in the development of atopy and subsequent atopic asthma.

A Major concern in case- control studies is the possibility of a false- positive association due to non homogeneity of the population structure. Indeed, one of the great advantages of family association study is the TDT which eliminates the possibility of spurious association due to population heterogeneity. The Transmission disequilibrium test (TDT) is widely used as a robust statistical method to test for genetic association due to linkage based upon analysis of parent-proband trios. However, Concerns about the potential of such designs to generate false positives as a result of poor matching between cases and controls has led to increasing use of family-based association analysis and in particular, the transmission disequilibrium test (TDT) which overcomes this theoretical problem. But we fail to show any preferential transmission of alleles from parents to affected children and the TDT in the present study has not shown evidence for linkage between VEGF polymorphisms and asthma. This result can be explained by the inheritance of asthma and atopy which is likely to be complex and to be confounded by relatively small contributions from a number of different genes. It is also likely to be confounded by partial penetrance, by asymptomatic carriers who have not yet been exposed to the relevant environmental hazard and by disease phenocopies. All of these factors are likely to be operating, thereby decreasing the power of linkage analyses, which assume that asthma and atopy exhibits Mendelian inheritance.

We acknowledge several limitations to our study. We only evaluated three functional polymorphisms of VEGF located in the same chromosome region 6p21.3, in order to perform haplotype analysis. It is possible that other functional polymorphisms in the VEGF gene may affect the association between these polymorphisms and asthma risk. Needless to say, absence of an association between these specific polymorphisms of VEGF in case control and family study does not eliminate the importance of VEGF polymorphisms and their implication in the severity process of the disease. Although we demonstrated an association of +936 C > T with the severity of the disease, the investigation of −634 G > C polymorphism should not be discarded and further studies considering this genetic marker should be performed to evaluate its relevance in asthma.

## Figures and Tables

**Table 1:** The clinical characteristics of our study samples.

		Controls	Asthmatics
	Number of subjects	224	210
	Age [mean(Standard deviation)]	9.5 (5–16)	11.5 (5–16)
	Mean age at the onset of asthma		4.6
	Sex ratio (girl/boy)	0.88	0.64
	Asthma in families	0	40%
	FEV 1*%, predicted mild asthma		86
	FEV 1%, predicted moderate asthma		70
	FEV 1%, predicted severe asthma		56
	Total IgE (IU/ml) atopic		640.04
	Total IgE (IU/ml) non atopic	62.30	58.8
Phenotypes associated	Rhinitis	0	14%
	Sinusitis	0	11%
	Dermatitis	0	3%
	RGO**	0	10%

* FEV1%, predicted: forced expiratory volume in 1s.

** RGO: gastro-oesophageal reflux.

**Table 2:** Frequency of alleles and distribution of genotypes of VEGF polymorphisms in asthmatics and controls subjects.

SNP		Genotypes				Alleles				
		Asthmatics n (%)	Controls n (%)	χ^2^	P value*	Asthmatics n (%)	Controls n (%)	χ^2^	P value**	OR(95% CI)
+936	CC	110(52.4)	132 (58.9)	1.9	0.3			2.0	0.15	0.80
	CT	80 (38.1)	75 (33.5)			**C** 300 (71.4)	339 (75.7)			(0.59–1.10)
	TT	20 (9.5)	17 (7.6)			**T** 120 (28.6)	109 (24.3)			
−634	GG	37 (17.6)	52 (23.2)	3.8	0.14			3.5	0.059	0.77
	GC	111 (52.9)	122 (54.5)			**G** 185 (44)	226 (50.4)			(0.59–1.02)
	CC	62 (29.5)	50 (22.3)			**C** 235 (56)	222 (49.6)			
−2549 −2567 del 18		30 (14.3)	24 (10.7)	5.3	0.07			0.3	0.56	1.08
(I/I)										(0.82–1.44)
(I/D)		96 (45.7)	127 (56.7)			**I** 156 (37.1)	175 (39.1)			
(D/D)		84 (40)	73 (32.6)			**D** 264 (62.9)	273 (60.9)			

**Table 3:** Haplotypes frequency of VEGF gene polymorphisms in asthmatics and controls children.

Haplotypes	−634	936	18	Asthmatics n (%)	Controls n (%)	χ^2^	P value	OR (95% CI)
ht 1	**G**	**C**	**I**	136 (32.38)	163 (36.38)	1.54	0.2	
ht 2	**G**	**T**	**I**	15 (3.57)	11 (2.45)		NS	
ht 3	**G**	**C**	**D**	29 (6.9)	27 (6.02)		NS	
ht 4	**G**	**T**	**D**	5 (1.2)	25 (5.58)	12.52	0.0004	0.20 (0.07–0.57)
ht 5	**C**	**T**	**I**	5 (1.2)	1 (0.22)		NS	
ht 6	**C**	**C**	**D**	135 (32.14)	149 (33.25)		NS	
ht 7	**C**	**T**	**D**	95 (22.61)	72 (16.07)	5.98	0.014	1.53 (1.07–2.18)
**Haplotypes combinations**								
1/1	GG	CC	II	20	23		<10^−3^	
1/2	GG	CT	II	5	0			
1/3	GG	CC	ID	7	4			
1/6	GC	CT	II	5	1			
2/3	GG	CT	ID	4	19			
2/4	GG	TT	ID	1	6			
3/5	GC	CC	ID	43	66			
3/6	GC	CT	ID	32	27			
3/8	GC	CT	DD	22	23			
4/6	GC	TT	ID	9	5			
5/5	CC	CC	DD	40	39			
7/8	CC	CT	DD	12	5			
8/8	CC	TT	DD	10	6			

**Table 4:** Distribution of differents genotypes and alleles for the VEGF polymorphisms in the atopic and non atopic group of asthmatics children.

SNPs		Genotypes				Alleles				
		Atopics Asthmatics n (%)	Non Atopics n (%)	χ^2^	P value*	Atopics Asthmatics n (%)	Non Atopics n (%)	χ^2^	P value**	OR (95% CI)
936 CC	CC	71 (51.1)	39 (54.9)	0.3	0.8			0.13	0.7	0.92
	CT	55 (39.6)	25 (35.2)			**C** 197 (70.9)	103 (72.5)			(0.57–1.84)
	TT	13 (9.3)	7 (9.9)			**T** 81 (29.1)	39 (27.5)			
−634	GG	23 (16.5)	14 (19.7)	3.8	0.14			0.5	0.4	1.17
	GC	80 (57.5)	31 (43.7)			**G** 126 (45.3)	59 (41.5)			(0.76–1.79)
	CC	36 (25.9)	26 (36.6)			**C** 152 (54.7)	83 (58.5)			
−2549 −2567 del 18		20 (14.4)	10 (14.1)	1.9	0.36			0.8	0.3	0.82
	(I/I)									(0.53–1.28)
	(I/D)	59 (42.4)	37 (52.1)			**I** 99 (35.6)	57 (40)			
	(D/D)	60 (43.2)	24 (33.8)			**D** 179 (64.4)	85 (59.9)			

**Table 5:** The distribution of VEGF polymorphism according to the asthma severity.

	Genotypes											
	+936 C > T				−634 G > C				−2549 −2567 del 18			
	CC	CT	TT	P value	GG	CG	CC	P value	II	ID	DD	P value
Intermittent and mild n (%)	68 (48.6)	65 (46.4)	7 (5)	0.0001	21 (15)	77 (55)	42 (30)	0.3	14 (10)	70 (50)	56 (40)	0.02
Moderate and Severe n (%)	42 (60)	15 (21.4)	13 (18.6)		16 (22.9)	34 (48.6)	20 (28.6)		16 (22.9)	26 (37.1)	28 (40)	

**Table 6:** Results of the transmission disequilibrium test (TDT) for the VEGF polymorphisms.

Alleles	Transmitted	Non transmitted
−634 G	50	60
−634 C	60	50
+936 C	73	63
+936 T	63	73
−2549 I 18(bp)	64	78
−2549 D 18(bp)	78	64

## References

[b1-grsb-2008-089] Asai K Kanazawa H Otani K 2002 Imbalance between vascular endothelial growth factor and endostatin levels in induced sputum from asthmatic subjects J. Allergy Clin. Immunol 110 571 5 12373263 10.1067/mai.2002.127797

[b2-grsb-2008-089] Asai K Kanazawa H Kamoi H 2002 Increased levels of vascular endothelial growth factor in induced sputum in asthmatic patients Clin. Exp. Allergy 33 595 9 12752587 10.1046/j.1365-2222.2003.01576.x

[b3-grsb-2008-089] Awata T Inoue K Kurihara S 2002 A common polymorphism in the 50-untranslated region of the VEGF gene is associated with diabetic retinopathy in type 2 diabetes Diabetes 51 1635 9 11978667 10.2337/diabetes.51.5.1635

[b4-grsb-2008-089] Boiardi L Casali B Nicoli D 2003 Vascular endothelial growth factor gene polymorphisms in giant cell arteritis J. Rheumatol 30 2160 4 14528511

[b5-grsb-2008-089] Chetta A Zanini A Foresiz A 2005 Vascular endothelial growth factor up-regulation and bronchial wall remodelling in asthma Clin. Exp. Allergy 35 1437 2 16297139 10.1111/j.1365-2222.2005.02360.x

[b6-grsb-2008-089] Feltis BN Wignarajah D Zheng L 2006 Increased vascular endothelial growth factor and receptors: relationship to angiogenesis in asthma Am. J. Respir. Crit. Care Med 173 1201 7 16528018 10.1164/rccm.200507-1105OC

[b7-grsb-2008-089] Ferrara N Houck K Jakeman L 1992 Molecular and biological properties of the vascular endothelial growth factor family of proteins Endocr Rev 13 18 32 1372863 10.1210/edrv-13-1-18

[b8-grsb-2008-089] Ferrara N 1999 Molecular and biological properties vascular endothelial growth factor J. Mol. Med 77 527 3 10494799 10.1007/s001099900019

[b9-grsb-2008-089] Ferrara N Gerber HP LeCouter J 2003 The biology of VEGF and its receptors Nat. Med 9 669 6 12778165 10.1038/nm0603-669

[b10-grsb-2008-089] Han SW Kim GW Seo JS 2004 VEGF Gene polymorphisms and susceptibility to rheumatoid arthritis Rheumatology 43 1173 7 15213335 10.1093/rheumatology/keh281

[b11-grsb-2008-089] Hoshino M Takahashi M Aoike N 2001 Expression of vascular endothelial growth factor, basic fibroblast growth factor and angiogenin immunoreactivity in asthmatic airways and its relationship to angiogenesis J. Allergy Clin. Immunol 107 295 1 11174196 10.1067/mai.2001.111928

[b12-grsb-2008-089] Jacobs EJ Feigelson HS Bain EB 2006 Polymorphisms In the vascular endothelial growth factor gene and breast cancer in the Cancer Prevention Study II cohort Breast Cancer Research 8 R.22 10.1186/bcr1400 PMC1557725 16613616

[b13-grsb-2008-089] Kanazawa H Hirata K Yoshikawa J 2002 Involvement of vascular endothelial growth factor in exercise induced bronchoconstriction in asthmatic patients Thorax 57 885 8 12324676 10.1136/thorax.57.10.885 PMC1746203

[b14-grsb-2008-089] Klagsbrun M D’Amore P 1996 Vascular endothelial growth factor and its receptors Cyt Growth Factor Rev 7 259 0 10.1016/s1359-6101(96)00027-5 8971481

[b15-grsb-2008-089] Kuwano K Bosken CH Pare PD 1993 Small airways dimensions in asthma and in chronic obstructive pulmonary disease Am. Rev. Respir. Dis 148 1220 5 8239157 10.1164/ajrccm/148.5.1220

[b16-grsb-2008-089] Krippl P Langsenlehner U Renner W 2003 A common 936 C/T gene polymorphism of vascular endothelial growth factor is associated with decreased breast cancer risk Int. J. Cancer 106 468 471 12845639 10.1002/ijc.11238

[b17-grsb-2008-089] Lambrechts D Storkebaum E Morimoto M 2003 VEGF is a modifier of amyotrophic lateral sclerosis in mice and humans and protects motoneurons against ischemic death Nat. Genet 34 383 394 12847526 10.1038/ng1211

[b18-grsb-2008-089] Lee YC Lee HK 2001 Vascular endothelial growth factor in patients with acute asthma J. Allergy Clin. Immunol 107 1106 11398093 10.1067/mai.2001.115628

[b19-grsb-2008-089] Lee CG Link H Baluk P 2004 Vascular endothelial growth factor (VEGF) induces remodeling and enhances TH2-mediated sensitization and inflammation in the lung Nat. Med 10 1095 3 15378055 10.1038/nm1105 PMC3434232

[b20-grsb-2008-089] Li X Wilson JW 1997 Increased vascularity of the bronchial mucosa in mild asthma Am. J. Respir. Crit. Care Med 156 229 233 9230753 10.1164/ajrccm.156.1.9607066

[b21-grsb-2008-089] Lutty GA McLeod DS Merges C 1996 Localization of vascular endothelial growth factor in human retina and choroid Arch. Ophthalmol 114 971 7 8694733 10.1001/archopht.1996.01100140179011

[b22-grsb-2008-089] Maniscalco WM Watkins RH D’Angio CT 1997 Hyperoxic Injury decreases alveolar epithelial cell expression of vascular endothelial growth factor (VEGF) In neonatal rabbit lung Am. J. Respir. Cell. Mol. Biol 16 557 67 9160838 10.1165/ajrcmb.16.5.9160838

[b23-grsb-2008-089] Neufeld G Cohen T Gengrinovitch S 1999 Vascular endothelial growth factor (VEGF) and its receptors Faseb J 13 9 22 9872925

[b24-grsb-2008-089] Papazoglou D Galazios G Koukourakis MI 2004 Association of −634G/C And 936C/T Polymorphisms of the vascular endothelial growth factor with spontaneous preterm delivery Acta. Obstet. Gynecol. Scand 83 461 5 15059159 10.1111/j.0001-6349.2004.00403.x

[b25-grsb-2008-089] Renner W Kotschan S Hoffmann C 2000 A common 936 C/T mutation in the gene for vascular endothelial growth factor is associated with vascular endothelial growth factor plasma levels J. Vasc. Res 37 443 8 11146397 10.1159/000054076

[b26-grsb-2008-089] Salvato G 2001 Quantitative and morphological analysis of the vascular bed in bronchial biopsy specimens from asthmatic and non-asthmatic subjects Thorax 56 902 6 11713351 10.1136/thorax.56.12.902 PMC1745985

[b27-grsb-2008-089] Shahbazi M Fryer AA Pravica V 2002 Vascular endothelial growth factor gene polymorphisms are associated with acute renal allograft rejection J. Am. Soc. Nephrol 13 260 4 11752046 10.1681/ASN.V131260

[b28-grsb-2008-089] Spielman RS McGinnis RE Ewens WJ 1993 Transmission test for linkage disequilibrium: the insulin gene region and insulin- dependent diabetes mellitus (IDDM) Am. J. Hum. Genet 52 506 8447318 PMC1682161

[b29-grsb-2008-089] Vincenti V Cassano C Rocchi M 1996 Assignment of the vascular endothelial growth factor gene to human chromosome 6p21.3 Circulation 93 1493 8608615 10.1161/01.cir.93.8.1493

[b30-grsb-2008-089] Watson CJ Webb NJ Bottomley MJ 2000 Identification of polymorphisms within the vascular endothelial growth. *Acta. Obstet. Gynecol. Scand*. 83 (2004) factor (VEGF) gene: correlation with variation in VEGF protein production Cytokine 12 1232 5 10930302 10.1006/cyto.2000.0692

[b31-grsb-2008-089] wjst M fischer G Immervoll T 1999 A genome wide search for linkage to asthma. German asthma genetics group Genomics 58 1 8 10333435 10.1006/geno.1999.5806

[b32-grsb-2008-089] Yancopoulos GD Davis S Gale NW 2000 Vascular-specific growth factors and blood vessel formation Nature 407 242 8 11001067 10.1038/35025215

[b33-grsb-2008-089] Yang B Cross DF Ollerenshaw M 2003 Polymorphisms of the vascular endothelial growth factor and susceptibility to diabetic microvascular complications in patients with type 1 diabetes mellitus J. Diabetes Complications 17 1 6 12505748 10.1016/s1056-8727(02)00181-2

